# Prognostic Role of Global DNA Methylation in Renal Cancer Reveals Decitabine Treatment Benefit

**DOI:** 10.2174/0109298673342146250217105350

**Published:** 2025-03-12

**Authors:** Wei Wu, Bin Huang, Peng Xia, Quanzhong Liu, Jin Yi, Ruohan Zhang, Qianghu Wang

**Affiliations:** 1 School of Biological Science & Medical Engineering, Southeast University, Nanjing, Jiangsu, 210000, China;; 2 Department of Bioinformatics, Nanjing Medical University, Nanjing, Jiangsu, 211166, China;; 3 Onkocare Life Technology (Suzhou) Company, Suzhou, Jiangsu, 215153, China;; 4 Collaborative Innovation Center for Personalized Cancer Medicine, Nanjing Medical University, Nanjing, Jiangsu, 211166, China;; 5 Jiangsu Institute of Cancer Research, Jiangsu Cancer Hospital, The Affiliated Cancer Hospital of Nanjing Medical University, 210002, Nanjing, China;; 6 The First Affiliated Hospital of Nanjing Medical University, Nanjing, 210029, China

**Keywords:** Renal cancer, global DNA methylation, prognosis, decitabine, biomarkers, tumor phenotype

## Abstract

**Background:**

Renal cancer presents a significant global health challenge due to its rising incidence and mortality rates. Often undetected in early stages, it complicates diagnosis and treatment. Current therapies face resistance and limited effectiveness, especially in advanced stages. The diverse subtypes of renal cancer highlight the need for new biomarkers and risk assessment tools for targeted treatments.

**Objective:**

This study aims to assess the prognostic significance of global DNA methylation (GM) levels in renal cancer, identify new biomarkers, and evaluate the therapeutic potential of the DNA methyltransferase inhibitor decitabine.

**Methods:**

Data on RNA sequencing, gene mutations, DNA methylation, and clinical outcomes were collected from TCGA and GEO databases. We calculated global DNA methylation scores (GMS) and categorized patients into high, intermediate, and low GMS groups. Survival analysis and genomic analyses were conducted to explore the relationships between GMS, clinical outcomes, and tumor characteristics.

**Results:**

Higher GMS was identified as an independent prognostic factor associated with worse outcomes in renal cancer. Patients with elevated GMS showed increased mutations, copy number variations, and a more aggressive tumor phenotype. Treatment with decitabine was observed to reduce tumor hypermethylation and downregulate cell cycle pathway activity, indicating potential therapeutic benefits.

**Conclusion:**

Global DNA methylation plays a significant role in renal cancer prognosis. GMS may serve as valuable biomarkers for prognosis and personalized treatment strategies. Decitabine shows potential efficacy for high GMS patients, particularly through its impact on cell cycle regulation, underscoring the importance of personalized approaches in cancer treatment.

## 1 INTRODUCTION

Renal cancer presents a significant global health burden, as evidenced by its rising incidence and mortality. In 2020, it accounted for an estimated 179,368 deaths worldwide, with a mortality rate of 1.8 per 100,000 individuals [1]. This trend is further highlighted by a 2019 study showing 371,700 new cases globally, with an age-standardized incidence rate of 4.6 per 100,000 [2]. Due to the silent nature of its early stages, renal cancer is often diagnosed late, which poses significant challenges for treatment [1, 3, 4]. The current treatment landscape for renal cancer is fraught with challenges, including resistance to conventional therapies and limited options for advanced-stage patients [5-7]. The diverse subtypes of renal cancer, primarily clear cell, papillary, and chromophobe carcinoma, underscore the absence of unified criteria for precise risk stratification [8-13]. Thus, there is a pressing need for the development of novel biomarkers and evaluation methodologies to improve risk assessment accuracy and to guide targeted therapeutic and management strategies, ultimately enhancing patient outcomes and quality of life [14].

CpG island methylation, which affects over half of human genes, is crucial in the development of cancer [15, 16]. Research has shown that a subset of these genes, governed by the Polysomy-Repressive Complex 2, undergo DNA hypermethylation and become inactive in cancerous states. This alteration influences critical pathways, such as the Epithelial-Mesenchymal Transition (EMT) and the TNFα-associated inflammatory response [17]. In renal cancer research, an analysis of 117 renal cell carcinoma (RCC) patients and 89 corresponding non-cancerous tissue samples was conducted. This investigation revealed a significant correlation between the increased methylation of the CpG island in GATA5 and increased metastasis in RCC patients, alongside a reduction in the disease-free progression period [18].

Furthermore, a more frequent occurrence of RASSF1A promoter hypermethylation was observed in grade 3 RCC patients, suggesting a worse prognosis [19].

Additionally, the study identified the hypermethylation of the SLC16A3 promoter, which controls MCT4, as an independent prognostic indicator for patients [20]. Despite these findings, the focus on individual genes and subtypes has led to a narrow perspective. The overlooked roles of other genes and biological processes, along with a gap in understanding gene interactions and intricate regulatory mechanisms, pose significant challenges in advancing therapeutic strategies. Few studies have investigated renal cancer from the perspective of the CpG Island Methylator Phenotype (CIMP). Using hierarchical clustering based on methylation β values at individual chromosomal positions from methylation arrays, researchers have identified a cluster of CIMP renal cancer patients with poor prognosis [21, 22]. However, these studies have identified only a few CIMP samples, with the majority being non-CIMP samples that lack prognostic guidance indicators. Additionally, they have not addressed the continuity of high global methylation levels underlying the CIMP phenotype, which may hold significant prognostic value for non-CIMP patients as well.

The current treatment landscape for renal cancer is diverse [23-25]. Surgery remains the preferred method for early-stage renal cancer, but most patients are diagnosed in advanced stages [26]. Chemotherapy has limited efficacy in renal cancer treatment due to the insensitivity of cancer cells to chemotherapeutic drugs. Immunotherapy, which activates the patient's immune system to fight tumor cells, is not universally applicable to all patients, and identifying those who will benefit is challenging, compounded by high costs. Targeted therapy, using drugs that specifically target biological markers in renal cancer cells, effectively inhibits tumor growth and spread. Common targeted drugs include inhibitors of the Vascular Endothelial Growth Factor (VEGF) and the mTOR pathway [27]. While targeted therapy has shown significant effectiveness in renal cancer, it may come with side effects, drug resistance, high costs, and limited applicability. Decitabine, a DNA methylation inhibitor, offers a potential global therapeutic action by reactivating silenced tumor suppressor genes and may enhance the immune system's ability to recognize tumors [28]. As it acts on DNA methylation, a common cellular mechanism, Decitabine might be effective against various types of cancer cells, especially those exhibiting abnormal methylation levels [29-32]. However, the specific effectiveness of Decitabine in treating renal cancer requires further research and validation.

Our study demonstrates that the average methylation level of CpG islands, indicative of GM, is an independent prognostic indicator in renal cancer. We found that higher GMS is associated with less favorable prognoses, suggesting that GMS is a potential novel biomarker for prognostic evaluation and treatment planning in renal cancer. Furthermore, patients with hypermethylation exhibited increased mutation rates and a higher prevalence of copy number variations. Tumor cells from these patients displayed enhanced proliferative potential and elevated tumor stem cell markers. Our research also underscores the potential therapeutic utility of Decitabine, a DNA methyltransferase inhibitor, particularly in patients with elevated GMS. This aligns with the growing emphasis on personalized medicine in cancer care, highlighting the need for targeted therapeutic interventions for effective treatment. This study significantly deepens our insight into renal cancer, especially in the context of epigenetic changes. It opens avenues for further exploration of the complex relationship between methylation patterns and treatment outcomes, potentially informing the development of innovative cancer therapies.

## 2 METHODS

### 2.1 Data Collection

RNA-seq, copy number variation, gene mutation, and DNA methylation, along with clinical and survival data, were obtained from the TCGA database (https://xenabrowser.net/datapages/). Additionally, methylation array data from GSE198673 [33] and GSE146179 [34], as well as transcriptome microarray sequencing data from GSE142381 [34], were acquired from the Gene Expression Omnibus (GEO) database (https://www.ncbi.nlm.nih.gov/geo/). TCGA generated these datasets using standardized protocols to ensure high data quality and consistency. RNA-seq data were produced using Illumina sequencing platforms, offering comprehensive gene expression profiles. DNA methylation data, mainly at CpG islands, were obtained *via* the Infinium HumanMethylation450 BeadChip array, providing high-resolution methylation measurements across the genome. CNV data were derived from SNP array platforms and processed with the GISTIC algorithm to identify significant copy number changes, both focal and broad, which are common in renal cancer and can influence tumor development. Gene mutation data were generated through whole-exome sequencing (WES) using Illumina platforms, allowing for the detection of somatic mutations with high sensitivity. Standardized pipelines, such as MuTect, were used for variant calling to accurately identify single nucleotide variants (SNVs) and small insertions/deletions (indels).

### 2.2 The Global DNA Methylation Score

We calculated the global methylation level for each TCGA renal cancer patient using data from the 450K methylation array. First, we processed the data with the ChAMP (v2.30.0) package, applying the champ.filter function to remove low-quality probes and probes located on sex chromosomes. We then identified probes located within CpG islands according to the hg19 genome reference. For each patient *P_i_*, we defined the set of relevant probes as *G_i_*, where each probe *g* ϵ *G* has a corresponding beta value *βi*.

The global methylation level *S_i_* for each patient *P_i_* was calculated using the following formula:


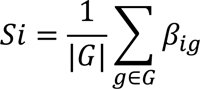


where |*G*| is the total number of CpG probes located on all CpG islands and *β_ig_* represents the methylation level of probe *g* for the patient *P_i_*.

Based on these global methylation scores *S_i_*, patients were divided into three groups: the top third with the highest *S_i_* values were classified as high global methylation score (HS), the bottom third as low global methylation score (LS), and the remaining third as intermediate global methylation score (IS). A summary of this categorization is provided in Supplementary Table **S1**.

### 2.3 Survival Analysis

Survival analysis was performed using the Kaplan-Meier method, focusing on OS and PFI as endpoints. Samples were categorized based on GMS, and inter-group comparisons were performed using the log-rank test. Survival curves were plotted using the survminer (v0.4.9) R (v4.3.1) package. Additionally, Cox proportional hazards regression analysis was employed to calculate the hazard ratio (HR). Results were visualized using the gg-forest (v3.5.5) function from the survival R package (Fig. **S1**).

### 2.4 Genomic Analysis

CNV and gene mutation data were obtained from the TCGA database (https://xenabrowser.net/datapages/). DNA mutation signatures data was sourced from Wang’s study [35]. We employed Fisher's exact test to identify different mutations between patients with HS and LS. We generated a hotspot mutation plot using the maftools (v2.16.0) R package. The CNV score for each sample was calculated by summing the squares of the copy number states for each gene (*e.g.*, -1 for loss, 0 for normal, and 1 for amplification) to assess CNV. We utilized Gistic2 (v2.0.23) [36] to identify and visualize copy number alterations (CNAs) between patients with HS and LS. The reference genome used was hg19. Amplification events were defined as scores greater than 0 with a q-value less than 0.05, while deletion events were classified as scores less than 0 with a q-value below 0.05.

### 2.5 Differential Gene Analysis

In comparing gene expression between HS and LS patients, genes identified by the Wilcoxon test with *p*-values below 0.05 and a fold change greater than 1.5 were classified as having high expression in HS patients. In contrast, those with *p*-values below 0.05 and a fold change less than 0.67 were classified as having low expression in HS patients.

### 2.6 Gene Set Enrichment Analysis

Gene functional analysis was performed by Enrcihr (https://maayanlab.cloud/Enrichr/) [37]. GSEA was performed utilizing clusterProfiler (v4.8.2) [38]. Gene sets corresponding to signaling pathways from the cancer-related hallmark dataset were downloaded from the msigDB database.

### 2.7 Stemness Analysis

Malta *et al.* [39] developed a new stemness index to assess cancer dedifferentiation, using a one-class logistic regression algorithm to derive transcriptomic and epigenetic features from non-transformed pluripotent stem cells and their differentiated progeny. We applied the stemness scores from their transcriptomic analysis of the TCGA samples to evaluate stemness levels in patients with varying GMS degrees.

### 2.8 Transcription Factor Activity Analysis

We utilized the decoupleR (v2.6.0) [40] R package to calculate the activation scores of transcription factors in each renal cancer sample obtained from the TCGA database. This algorithm analyzes gene expression data to assess the activity of transcription factors based on their target gene profiles. For each sample, we input the normalized expression levels of genes associated with specific transcription factors into decoupleR. The algorithm then applies statistical models to infer the activation states of these transcription factors, providing a quantitative score that reflects their activity within the tumor environment.

### 2.9 Assessment of Immune Cell Infiltration and Immune Checkpoint Expression

Using the CIBERSORT [41] algorithm, we analyzed immune cell infiltration levels based on the transcriptomic expression data from renal cancer. This analysis included M1/M2 macrophages, plasma cells, activated mast cells, and T regulatory cells. We compared the expression of immune checkpoint-associated inhibitory and activating genes between the HS and LS groups.

### 2.10 Decitabine Treatment Data Analysis for Renal Cancer Cells

To assess the impact of Decitabine treatment on global methylation in renal cancer cells, we obtained methylation data from the GSE198673 (850K) and GSE146179 (450K) datasets. We then calculated the global methylation levels of each sample using the previously described method. Additionally, to evaluate the biological effects of Decitabine treatment, we downloaded transcriptomic data from GSE142381. We compared gene expression levels before and after treatment, selecting genes with an average post-treatment expression decrease of more than 0.5 compared to pre-treatment levels. Functional enrichment analysis of down-regulated genes after therapy was then conducted using hallmark gene sets from the MsigDB database and transcription factor targets from the ENCODE and ChEA databases *via* the enrichr (https://maayanlab.cloud/Enrichr/) tool.

## 3 RESULTS

### 3.1 GM is an Independent Prognostic Factor for Renal Cancer

**Fig. (1):**
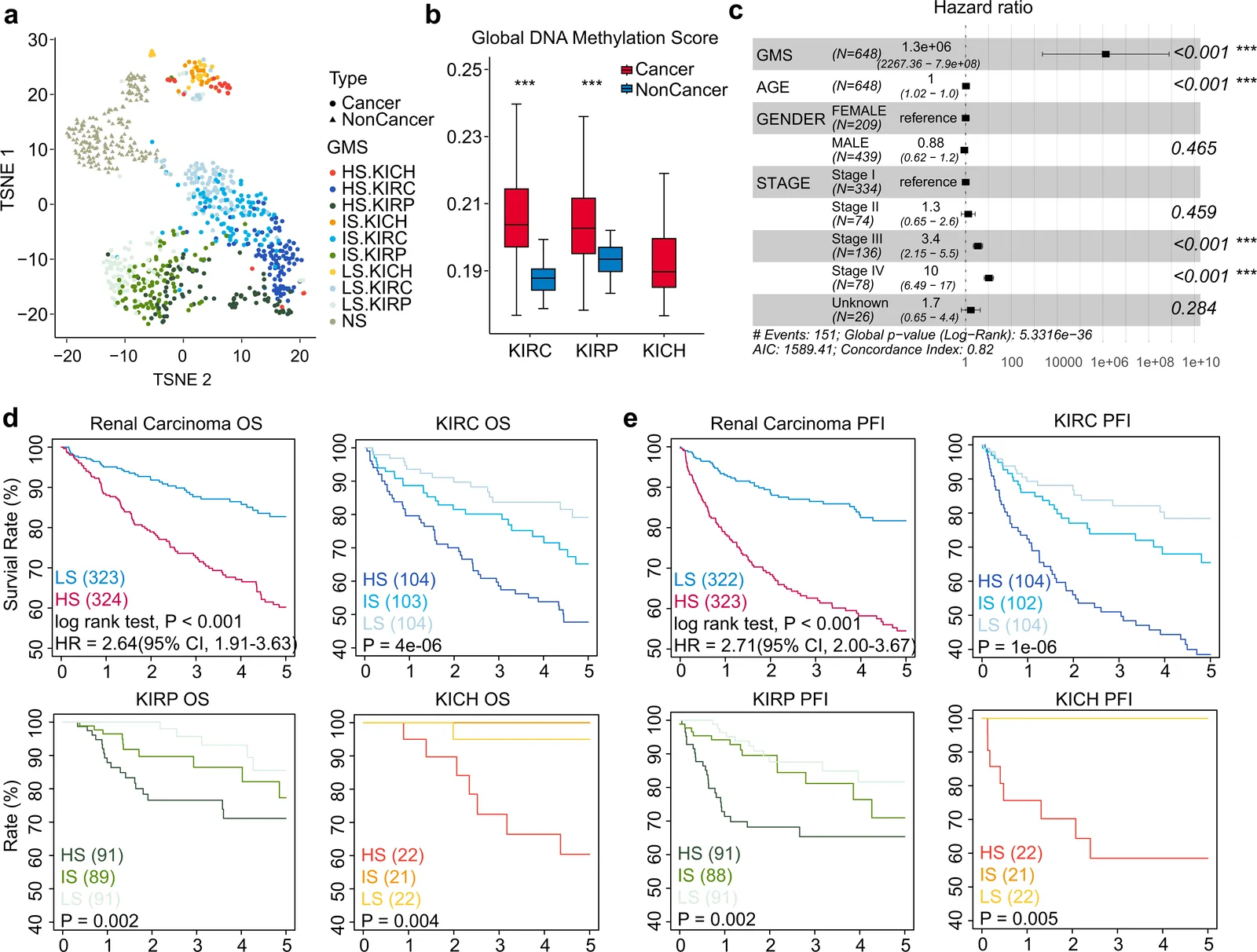
Prognostic role of GMS in renal patients. (**a**) TSNE dimensionality reduction plot of the GMS for each sample, HS represents high global DNA methylation, IS represents intermediate global DNA methylation, LS represents low global DNA methylation, NS represents global DNA methylation of non-tumor counterparts. (**b**) Comparison of GMS between tumor and non-tumor samples across the three subtypes. *p<0.05, **p<0.01, ***p<0.001. (**c**) Multifactorial survival analysis forest plot, where STAGE represents clinical staging. *p<0.05, **p<0.01, ***p<0.001. (**d**, **e**) Kaplan-Meier analysis stratified by GMS above and below the median in the TCGA cohort of renal cancer patients. P represents the log-rank test *p*-value, and HR represents the hazard ratio. OS represents overall survival, and PFI represents progression-free survival.

The aberrant methylation of CpG islands within tumor cells leads to the widespread silencing of tumor suppressor genes, thereby fueling the progression and severity of the disease. To elucidate the significance of this methylation pattern in renal cancer, we calculated the GMS for 648 tumor samples across three renal cancer subtypes, including 311 kidney renal clear cell carcinoma (KIRC), 272 kidney renal papillary cell carcinoma (KIRP), 65 kidney chromophobe (KICH), and 200 adjacent non-tumor renal tissues, using the 450K methylation array from The Cancer Genome Atlas (TCGA). T-distributed Stochastic Neighbor Embedding (t-SNE) analysis revealed that the non-tumor counterparts of all three subtypes exhibited similar global methylation patterns, distinct from those observed in tumor samples. Additionally, within each subtype, patients displayed consistent global methylation profiles, with samples distributed across a spectrum of scores. Distinct GMS profiles were noted among the different subtypes (Fig. **1a**), with KIRC tumor tissues showing the highest GMS, followed by KIRP and KICH scoring the lowest. In contrast, all non-tumor tissues exhibited lower GMS than their tumor counterparts (Fig. **1b**). The level of global DNA methylation in patients presented a significantly higher survival hazard ratio (HR) than age, gender, and stage (HR=1300000, *p*<0.001, Fig. **1c**), indicating GM was an independent prognostic factor for renal cancer. Renal cancer patients with high global DNA methylation scores (HS) had significantly shorter overall survival (OS) compared to those with low scores (LS) (adjusted HR=2.64, 95% CI: 1.91-3.63, *p*<0.001). This trend was observed across the KIRC, KIRP, and KICH subtypes, with the GMS being particularly indicative of risk stratification in KIRP patients (Fig. **1d**). This trend was evident in predicting progression-free interval (PFI), with patients in the HS group being associated with significantly shorter PFIs across all renal cancer subtypes (adjusted HR=2.71, 95% CI: 2-3.67, *p*<0.001). This trend was observed within each subtype, and the GMS scores enabled a clearer separation of PFI durations among KIRC patients (Fig. **1e**). Notably, GMS enabled stratification of late-stage patients (stages 3 and 4) by Overall Survival (OS) and Progression-Free Interval (PFI). In Ricketts’ study, methylation array data were used to cluster renal cancer samples into three categories. Among these, cluster 1 was identified as the group with the poorest prognosis and included 10 CIMP samples. However, we found cluster 1 exhibits heterogeneity. By using GMS, risk stratification for KIRC and KIRP patients within cluster 1 was achievable (Fig. **S1**). These findings underscored the GMS as a potent prognostic indicator capable of delineating diverse risk levels and aiding in patient management, particularly for advanced renal patients. This advancement in precision medicine held promise for improved patient outcomes.

### 3.2 Patients with HS Exhibit Distinct Genomic Patterns Compared to Those with LS

**Fig. (2):**
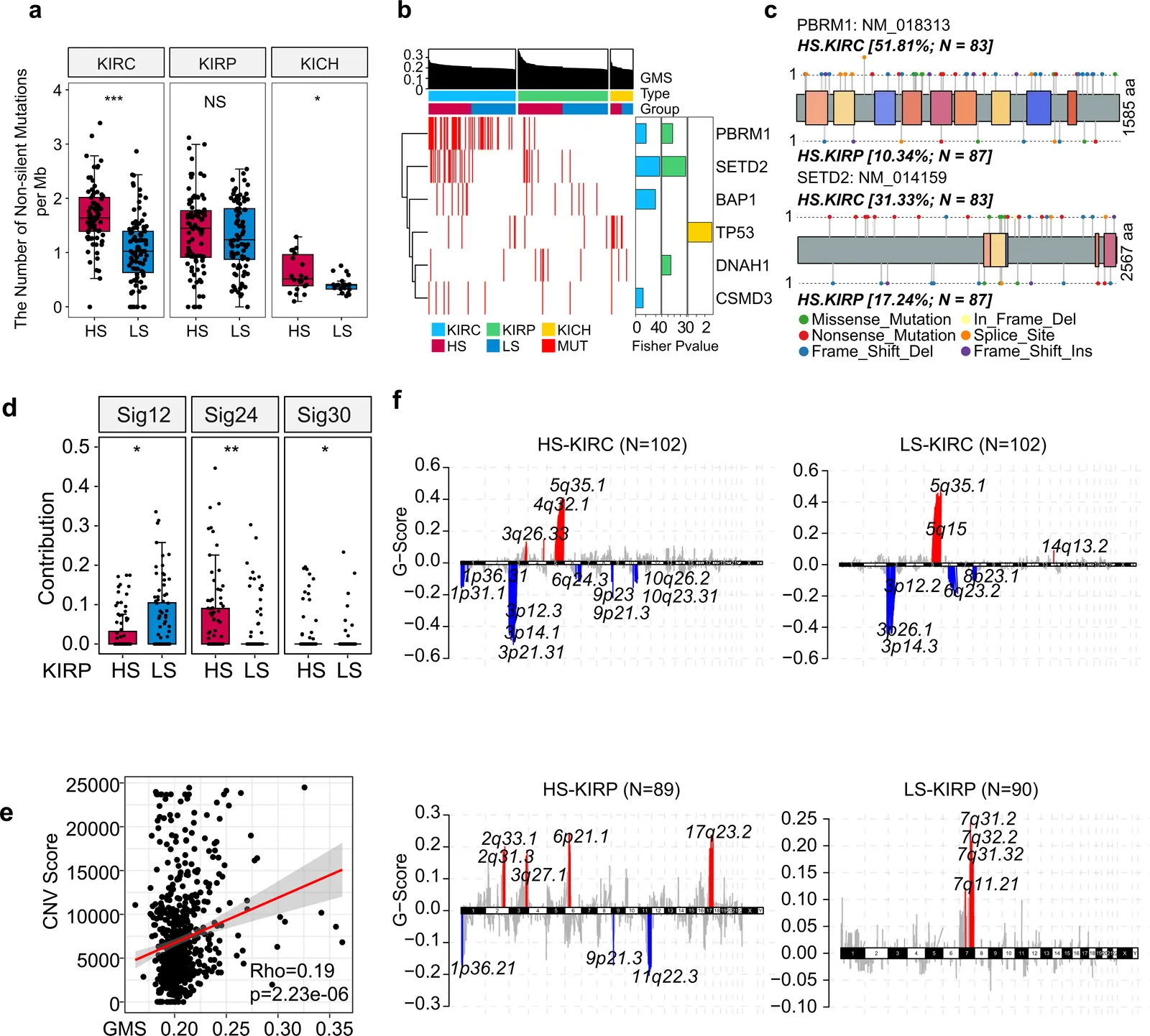
Genomic Alterations in renal patients with HS compared to those with LS. (**a**) Comparison of gene mutation frequencies. *p<0.05, **p<0.01, ***p<0.001. (**b**) Specific gene mutation differences between two groups. (**c**) Hot-spot mutation plot. (**d**) Frequency of mutation signatures occurring between patients with HS and LS. *p<0.05, **p<0.01, ***p<0.001. (**e**) Scatter plot of correlation analysis between CNV score and GMS. (**f**) Specific chromosomal variations between KIRC and KIRP patients with HS and LS.

DNA methylation has the potential to initiate genomic events, and to clarify the relationship between GM and genomic alterations in renal cancer patients, and we compared the incidence of genomic events between patients with HS and those with LS. Our results revealed a significantly higher tumor mutation burden (TMB) in KIRC and KICH patients with HS *versus* those with LS (Fig. **2a**). We also investigated the specific genomic mutation differences between the two groups, finding that PBRM1 and SETD2 mutations were more prevalent in KIRC and KIRP patients with HS. In contrast, TP53 mutations were more enriched in KICH patients (Fig. **2b**). Previous studies have indicated that PBRM1 loss is associated with resistance to immune checkpoint inhibitors in renal cancer [42]. High expression of PBRM1 can inhibit the growth of renal cancer cells [43]. Conversely, loss of function due to SETD2 mutations can promote the progression of malignant renal cancer cells through replication stress and impaired DNA repair [44]. Furthermore, a significant proportion of KIRC patients with HS exhibited PBRM1 mutations, with 51.81% (43/83) of these patients affected. In the KIRP subtype, 10.34% (9/87) of HS patients harbored PBRM1 mutations. For SETD2 mutations, 31.33% (26/83) of KIRC patients with HS were affected, while 17.24% (15/87) of KIRP patients with HS had SETD2 mutations. Notably, the mutation sites of PBRM1 and SETD2 did not reveal any specific hotspot mutations (Fig. **2c**). Mutation signatures are patterns of DNA changes that can indicate specific causes or exposures related to cancer. According to COSMIC, the sources of signatures 12 and 30 are currently unknown, while signature 24 has been linked to aflatoxin exposure. Signature 24 is found in some liver cancers and shows a strong mutation pattern associated with DNA damage from aflatoxin, which is repaired through transcription-coupled nucleotide excision repair. We examined the relationship between these mutation signatures and GMS scores, finding that signature 12 was more common in KIRP patients with low GMS scores. In contrast, signatures 24 and 30 appeared more frequently in patients with high GMS scores. This suggests that KIRP patients with higher GMS scores may have been exposed to aflatoxin (Fig. **2d**). Furthermore, we discovered a positive correlation between copy number variation (CNV) scores and GMS in patients, indicating that GMS is associated with genomic instability (Fig. **2e**). Specifically, amplifications at 3q and 4q, and deletions at 1p, 9p, and 10q were more enriched in KIRC patients with HS compared to those with LS. Similarly, amplifications at 2q, 3q, and 6p and deletions at 1p, 9p, and 11q were observed more enriched in KIRP patients with HS *versus* those with LS. Notably, KIRP patients with LS exhibited significant amplification in 7q compared to those with HS (Fig. **2f**). Collectively, these results suggested that GMS correlates with genomic variation events, potentially contributing to the onset and progression of renal cancer.

### 3.3 Patients with HS display Unique Transcriptomic Patterns Compared to those with LS

**Fig. (3):**
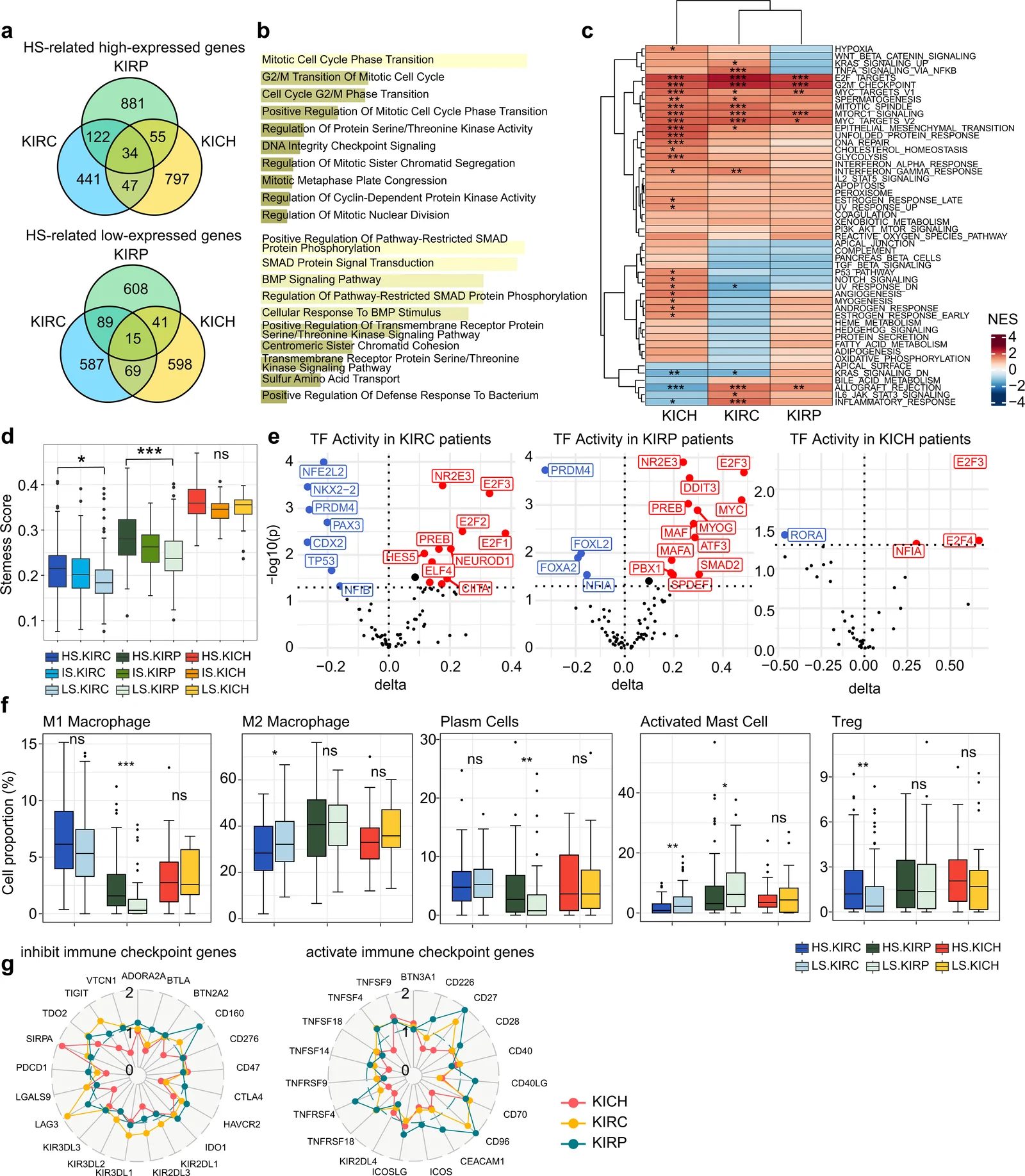
Transcriptomic Alterations in patients with HS compared to those with LS. (**a**) Venn diagram depicting the common differentially expressed genes between the HS and LS groups across KIRC, KIRP, and KICH patients. (**b**) Gene enrichment results for 34 upregulated genes in the HS group are shown above, and enrichment results for 15 downregulated genes in the HS group are shown below. (**c**) Heatmap of hallmark pathway scores. *p<0.05, **p<0.01, ***p<0.001. (**d**) Comparative analysis of tumor cell stemness scores among HS, IS, and LS groups. *p<0.05, **p<0.01, ***p<0.001. (**e**) Activated and inhibited transcription factors in renal cancer patients. (**f**) Comparative analysis of immune cell infiltration levels between HS and LS groups. *p<0.05, **p<0.01, ***p<0.001. (**g**) Differential expression analysis of immune checkpoint-related genes between patients with HS and LS. Immune inhibitory-related genes are listed on the left, while immune activating-related genes are listed on the right. Values represent the fold change in gene expression levels between the HS and LS groups.

DNA methylation in promoter regions exerts a profound influence on gene transcription, modulating the expression of genes that are crucial for cellular processes, development, and disease. To delve into the correlation between global methylation and transcriptional activity in renal cancer, we assessed gene expression between patients with HS and LS. We found that 34 genes were upregulated, and 15 genes were downregulated in patients with HS across the three renal cancer subtypes (Fig. **3a**, Supplementary Table **S2**). The upregulated genes were significantly enriched in pathways associated with the mitotic cell cycle phase transition, the G2/M transition of the mitotic cell cycle, and the G2/M phase transition. Conversely, the downregulated genes were significantly enriched in pathways related to the SMAD protein signaling pathway and BMP signaling pathways (Fig. **3b**). Similarly, gene set enrichment analysis (GSEA) uncovered the activation of E2F targets, G2M checkpoint pathways, and the MYC pathway across three subtypes (Fig. **3c**). The aberrant regulation of mitotic cell cycle phase transition and G2/M phase transition pathways may facilitate tumor cell proliferation and genomic instability, accelerating disease progression. A deeper understanding of these pathways’ mechanisms may lead to the identification of novel therapeutic targets for enhancing the prognosis of renal cancer patients.

Additionally, based on stemness index of TCGA samples calculated by Malta’s study [39], we observed a significant rise in tumor stem cell scores in KIRC and KIRP patients with HS compared to those with LS, indicating tumor cells of these patients may have a stronger resistance to chemotherapy (Fig. **3d**). Moreover, transcription factor analysis revealed the significant activation of E2F family transcription factors across all three subtypes as well (Fig. **3e**). Using transcriptomic data, we quantified immune cell infiltration proportions across the three subtypes. We observed a notable increase in M1 macrophages in KIRP patients with HS, while M2 macrophages predominated in KIRC patients with LS (Fig. **3f**).

**Fig. (4):**
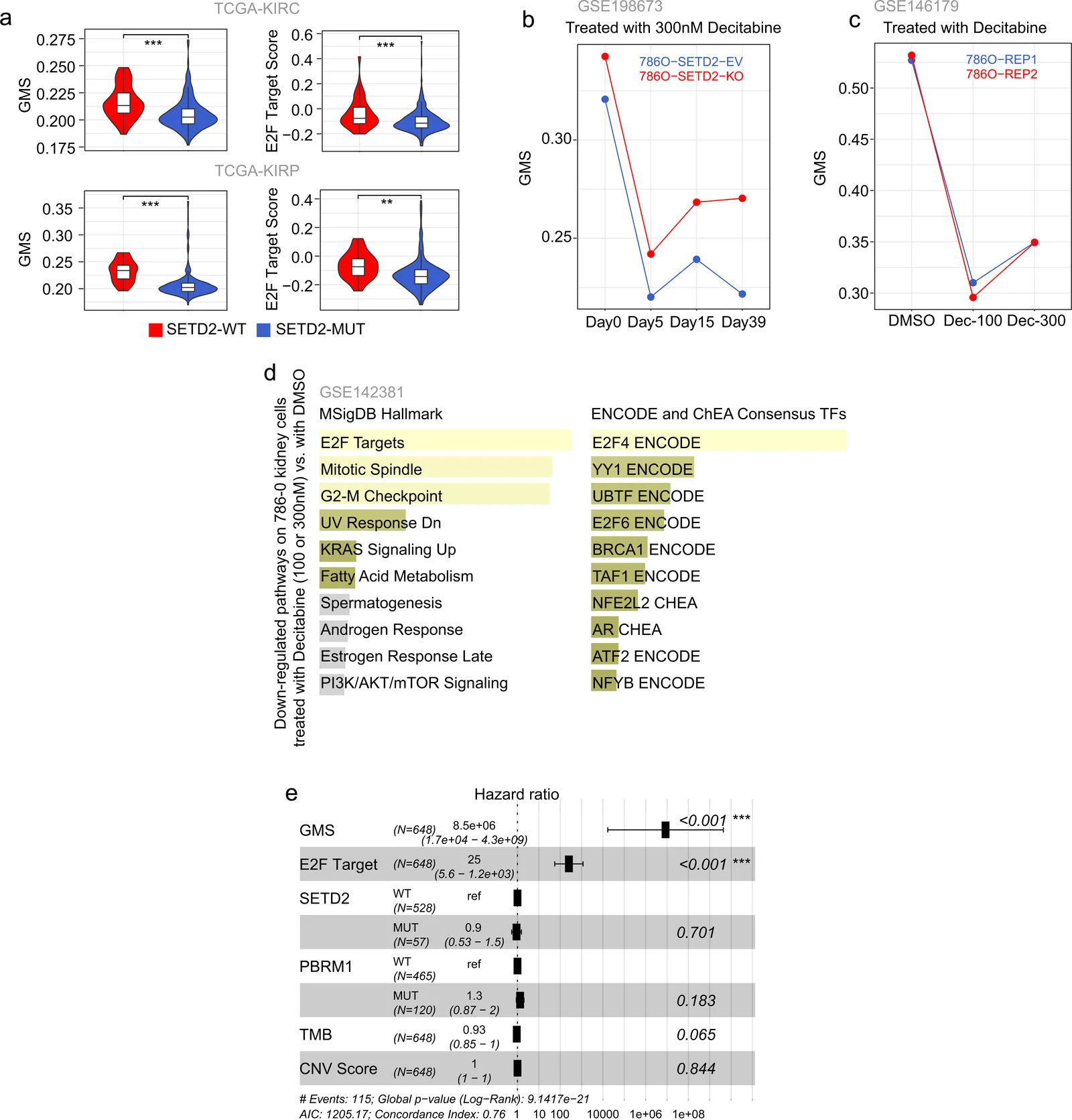
Decitabine Reduces Hypermethylation and Modulates Cell Cycle Pathways. (**a**) Comparison of GMS and E2F Target scores between samples with SETD2 mutations and SETD2 wild-type samples, red indicates SETD2 gene mutations, while blue indicates wild-type samples. *p<0.05, **p<0.01, ***p<0.001. (**b**) Detection of methylation levels at different time points in 786O cells (with and without SETD2 knockout) treated with 300nM Decitabine. (**c**) Effect of different concentrations of Decitabine on DNA methylation in 786O cells. (**d**) Functional enrichment analysis of genes inhibited by Decitabine in cells. (**e**) Cox regression analysis of gene mutation events related to OS.

Additionally, regulatory T cells exhibited significantly higher infiltration levels in KIRC patients with HS. Moreover, our analysis revealed that genes associated with inhibitory immune checkpoints are markedly upregulated compared to those linked with activating immune checkpoints in renal cancer patients. This discovery contributed to a deeper understanding of the immune mechanisms at play in renal cancer (Figs. **3g, 4**). This evidence indicated that DNA hypermethylation may suppress the expression of genes related to activating immune niche while promoting the expression of inhibitory genes, thereby enabling tumor cells to evade immune surveillance.

### 3.4 Decitabine is a Potential Therapeutic Drug for Renal Cancer Patients

Decitabine, a DNA methyltransferase inhibitor commonly used for myelodysplastic syndromes and acute myeloid leukemia, has shown potential in renal cancer treatment through some clinical studies. Decitabine shows promise as a therapeutic option for renal cancer patients. The above Findings indicated that poor prognosis in these patients is linked to heightened cell cycle pathway activity and mutations in the SETD2 gene, especially in those with high GMS. We aimed to investigate whether SETD2 mutations contribute to maintaining high GMS levels in patients. We found that patients with SETD2 mutations exhibited higher GMS levels and increased activity in the E2F target pathway, which is associated with tumor cell proliferation and malignant progression. This increased pathway activity may account for the poorer prognosis observed in SETD2-mutant patients compared to those with wild-type SETD2. *in vitro*, studies demonstrated that SETD2 knockout 786-O renal cancer cell lines maintained higher GMS than their wild-type counterparts before decitabine treatment. Upon treatment with 300 nM Decitabine, GMS in these cells dropped to its lowest level by day five, though a slight rebound occurred by day fifteen. By day 39, SETD2 knockout cells still showed elevated GMS, while wild-type cells exhibited a significant decline, suggesting that SETD2 may play a role in maintaining high methylation levels.

Further studies by Aguirre and colleagues treated 786-O cell lines with varying concentrations of Decitabine, showing that lower concentrations (100 nM) produced no significantly different reduction in global methylation levels than 300 nM. This suggested that both 100 nM and 300 nM concentrations of Decitabine can effectively alter global methylation in renal cancer cells. To understand Decitabine's impact on biological pathways in renal cancer, we analyzed transcriptomic data from the GSE142381 dataset. Following treatment with 100 nM and 300 nM Decitabine, genes related to the E2F target and G2-M checkpoint pathways were notably downregulated (Supplementary Table **S3**), particularly the E2F4 transcription factor family. This indicates that Decitabine inhibits genes associated with cell proliferation, specifically the E2F target pathway. As expected, the suppression of E2F pathway activity alone was associated with lower prognostic risks compared to high methylation scores (HR = 8500000, *p* < 0.001). These results suggested that GMS was a significant factor affecting prognosis in renal cancer.

## 4 DISCUSSION

We conducted a comprehensive analysis of epigenetic, genomic, and transcriptomic profiles across three renal cancer subtypes, examining 648 tumor samples and 200 adjacent non-tumor tissues. This study sheds new light on GM patterns, transcriptome, and genomic alterations in renal cancer patients. Aberrant DNA methylation plays a pivotal role in driving tumor progression [45-47]. DNA methylation primarily occurs at CpG islands, which are in a low-methylation state under normal physiological conditions [48]. However, methylation levels increase as needed to participate in gene regulation. Aberrant methylation states, such as hypomethylation of oncogenes and hypermethylation of tumor suppressor genes, are pivotal events in carcinogenesis [49, 50]. Previous studies have underscored the pivotal role of DNA methylation in the pathogenesis of renal cancer, a disease characterized by a complex interplay of genetic and epigenetic changes [45, 51, 52]. Consequently, a quantitative indicator is essential for describing the progression and prognosis of kidney cancer. In this study, we developed an algorithm to quantify the average CpG Island DNA methylation level of each sample, identifying a common feature among the three renal cancer subtypes: patients with high methylation levels were closely associated with poor prognosis. Using our algorithm, we categorized all samples into HS and LS groups. DNA methylation is instrumental in determining genome structure and function, including regulating cell proliferation, differentiation, and coordinating gene expression [53]. In our study, patients in the HS group exhibited higher gene mutation frequencies and chromosomal instability, with PBRM1 and SETD2 showing the highest mutation frequencies. Moreover, the impact of DNA methylation on gene expression was particularly pronounced. In the HS group, cell cycle-related genes were significantly upregulated, particularly with activation in the G2M and E2F target signaling pathways. Our analysis revealed that renal cancer patients with poor prognosis exhibited elevated DNA methylation levels and enhanced cell cycle signaling as a defining feature. Treatment with the demethylating agent decitabine reduced GM levels in 786O cells, leading to a decrease in cell cycle activity.

DNA methylation is fundamental to the establishment and preservation of normal cellular functions, and its aberrant patterns in tumor cells contrast markedly with those observed in healthy cells. At the outset and throughout the progression of tumorigenesis, epigenetic modifications precede global alterations in DNA methylation [54, 55]. We calculated the GMS for each sample and we determined that tumor samples can be distinctly differentiated from adjacent non-tumor samples using these scores. Elevated DNA methylation is a prevalent characteristic of tumor tissues across the three subtypes, aligning with prior research [50]. Significantly, the prognosis based on GMS exhibits greater predictive power than traditional clinical analyses. Both overall and disease-free survival post-treatment are closely tied to GMS. Our algorithm surpasses existing predictive methods, such as transcriptome-based predictions utilizing multiple indicators [56], in terms of performance. In previous studies, only a few CIMP renal cancer samples have been identified. For other non-CIMP renal cancer samples, such as ChRCC, PRCC1, PRCC2, and CCRCC, no significant differences in survival have been observed. However, GMS has been shown to effectively stratify the survival risk for these samples [21]. Incorporating GMS detection into clinical diagnosis and treatment planning may assist in early intervention strategies and help anticipate treatment outcomes for patients.

Previous studies have established a positive association between the response to immune therapy and TMB in solid tumors [57-59]. However, the correlation between TMB and immune therapy response in RCC remains a subject of debate [60, 61]. Our study revealed that RCC patients with HS demonstrate a high incidence of gene mutations, which may contribute to their unfavorable prognosis. Furthermore, PBRM1 and SETD2 were identified as the two genes with the highest mutation frequencies. SETD2, functioning as an RNA polymerase II-associated histone methyltransferase responsible for H3K36me2 methylation, is one of the most frequently mutated chromatin modifier genes across various cancer types, with a mutation rate of 13% in clear cell renal cell carcinoma (ccRCC), predominantly manifesting as truncating mutations. Genomic profiling of primary ccRCC tumors has indicated a correlation between SETD2 mutations and metastasis [62-64]. Consistent with our observations, patients with SETD2 mutations exhibited high methylation scores and poor prognosis. Mutation signature 24 is linked to aflatoxin exposure, which is known to cause specific DNA damage patterns. In our study, the mutation frequency of feature 24 was significantly elevated in the HS subgroup, suggesting a possible higher exposure to aflatoxin in these patients.

Methylation of CpG islands within gene promoter regions can intensify the heterochromatinization of the promoter region, obstructing the access and binding of the transcription initiation complex and thereby enhancing the transcriptional repression of the gene [65, 66]. It has been documented that, under certain conditions, high methylation of promoter DNA hinders the binding of transcriptional repressor factors, potentially leading to the activation of downstream genes [67]. In samples with elevated methylation levels, we identified 34 genes that were upregulated across all three cancer types, which were involved in enhancing the activity levels of the Mitotic Cell Cycle Phase Transition and G2/M Transition of Mitotic Cell Cycle signaling pathways. In renal cell carcinoma, the abnormal activation or inactivation of the Mitotic Cell Cycle Phase Transition can result in premature or delayed entry of cells into mitosis, leading to chromosomal instability and abnormal cell division, thereby fostering tumor development and progression [68]. The transition from the G2 phase to the M phase is a pivotal step in mitosis. Enhanced signaling can increase the frequency and instability of cell division, further accelerating tumor development and dissemination. Moreover, an analysis of stemness revealed that patients with HS exhibited stronger tumor stemness, as tumor stem cells can continuously self-renew, sustaining tumor growth and progression. This suggests that these patients may be at a higher risk of recurrence and metastasis and may respond poorly to conventional treatment regimens. Furthermore, the activation of the E2F family of transcription factors was observed in the HS group. Members of the E2F family can regulate the G1/S phase transition of the cell cycle and DNA synthesis in the S phase, thereby promoting cell proliferation and growth. Previous studies have suggested that in renal cell carcinoma, the aberrant activation of the E2F family may impact the response of tumor cells to DNA damage, leading to reduced DNA repair capacity and increased cell survival [69].

Current treatment options for advanced renal cancer include chemotherapy, immunotherapy, and targeted therapies [23, 70-73]. While chemotherapy can offer temporary relief, tumor cells often quickly develop resistance [74, 75]. Immunotherapy is effective in only a subset of patients, and identifying responders is both challenging and costly [76]. Targeted therapies have shown promising results but may be limited by side effects and drug resistance. In our study, we observed that higher DNA methylation levels in renal cancer tumor tissues were linked to poorer prognosis. Decitabine, a DNA methyltransferase inhibitor, may have global anti-tumor effects by reactivating silenced tumor suppressor genes and potentially enhancing the immune system's ability to recognize tumors [77]. Weidng Han *et al.* found enhanced anti-tumor activity, cytokine production, and proliferation of Decitabine-treated CAR-T cells in *in vitro* and *in vivo* studies [78]. Consistent with these findings, our study demonstrates that decitabine effectively reduces DNA methylation and downregulates cell cycle-related genes in renal cancer cell lines, highlighting its potential as a therapeutic option for renal cancer. Notably, the combination of epigenetic therapy and immunotherapy is currently being evaluated in several clinical trials and has shown promise in kidney cancer [77]. This combination could represent a valuable strategy in the treatment of renal cancer, warranting further exploration.

## CONCLUSION

In conclusion, our study contributes to the understanding of renal cancer by highlighting the prognostic value of the GMS biomarker, which may help guide personalized treatment strategies. Our findings suggest that the DNA methyltransferase inhibitor Decitabine reduces hypermethylation and modulates cell cycle pathways, supporting the potential of precision medicine in renal cancer treatment. This work also provides a foundation for further research into the relationship between methylation patterns and treatment responses, with the goal of developing more tailored and effective therapies for renal cancer patients. This approach offers new insights into renal cancer progression and advances the pursuit of targeted patient care.

## STUDY LIMITATIONS

As reflected in the abstract, the present study is primarily correlational and relies on bioinformatics analyses using data from the TCGA and GEO databases, without wet-lab experimental validation. Therefore, all findings should be interpreted as associative rather than causal as no causal relationships between global DNA methylation levels, clinical outcomes, or decitabine effects can be inferred from the current data. Further experimental studies are needed to validate the prognostic value of GMS and the therapeutic potential of decitabine in renal cancer.

## AUTHORS’ CONTRIBUTIONS

The authors confirm their contributions to the paper as follows: Q.W. and R.Z. designed the research; W.W. and B.H. analyzed the data; W.W. and P.X. wrote the manuscript. Q.L., J.Y. supervised the work and reviewed this manuscript. All authors read and approved the final manuscript.

## Data Availability

The data and supportive information are available within the article.

## LIST OF ABBREVIATIONS

GM: Global DNA Methylation
GMS: Global DNA Methylation Score
RCC: Renal Cell Carcinoma
VEGF: Vascular Endothelial Growth Factor
KIRC: Kidney Renal Clear Cell Carcinoma
KIRP: Kidney Renal Papillary Cell Carcinoma
KICH: Kidney Chromophobe
TCGA: The Cancer Genome Atlas
t-SNE: t-distributed Stochastic Neighbor Embedding
HS: High Global DNA Methylation Scores
IS: Intermediate Global DNA Methylation Scores
LS: Low Global DNA Methylation Scores
NS: Non-tumor Global DNA Methylation Scores
TMB: Tumor Mutation Burden
CNV: Copy Number Variation
GSEA: Gene Set Enrichment Analysis
ccRCC: Clear Cell Renal Cell Carcinoma
HR: Hazard Ratio
OS: Overall Survival
PFI: Progression-free Interval
CIMP: CpG Island Methylator Phenotype
WES: Whole-exome Sequencing
SNV: Single Nucleotide Variant
