# Supplementary material for: Prognostic Role of Global DNA Methylation in Renal Cancer Reveals Decitabine Treatment Benefit
Source: Curr Med Chem. 2025 Mar 12;33(18):3663–81. doi: 10.2174/0109298673342146250217105350 (PMC13555769; doi:10.2174/0109298673342146250217105350)
Supplement: Supplementary file 1 [file CMC-33-18-3663_SD1.zip › CMC-33-18-3663_SD1/BMS-CMC-2024-473 Suppl.pdf]

Supplementary Material

Prognostic Role of Global DNA Methylation in Renal Cancer Reveals Decitabine Treatment Benefit

Wei Wu[1,2,#], Bin Huang[1,2,#], Peng Xia[1,2,#], Quanzhong Liu[2], Jin Yi[3], Ruohan Zhang[2,4,*] and Qianghu Wang[1,2,4,5,6,*]

[1]School of Biological Science & Medical Engineering, Southeast University, Nanjing, Jiangsu, 210000, China; [2]Department of Bioinformatics, Nanjing Medical University, Nanjing, Jiangsu, 211166, China; [3]Onkocare Life Technology (Suzhou) Company, Suzhou, Jiangsu, 215153, China; [4]Collaborative Innovation Center for Personalized Cancer Medicine, Nanjing Medical University, Nanjing, Jiangsu, 211166, China; [5]The Affiliated Cancer Hospital of Nanjing Medical University, Jiangsu Cancer Hospital, Jiangsu Institute of Cancer Research, 210002Nanjing, China; [6]The First Affiliated Hospital of Nanjing Medical University, Nanjing, 210029, China

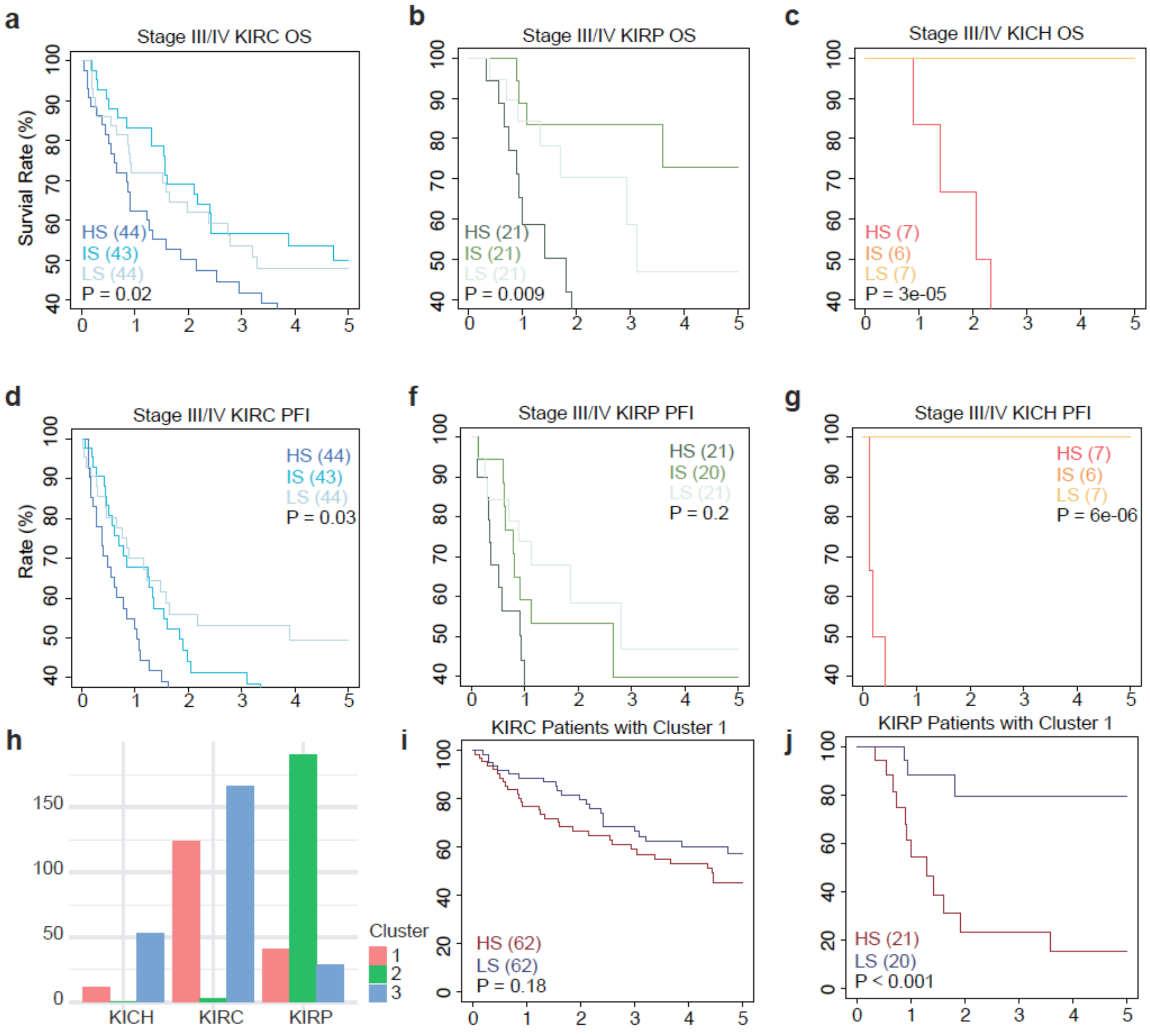

Fig. (S1). KM analysis of advanced kidney cancer patients graded according to GMS. (**a-g**) Kaplan-Meier analysis stratified by KIRC, KIRP, and above and below median GMS in advanced KICH patients. P represents the log-rank test p-value, and HR represents the hazard ratio. OS represents overall survival, and PFI represents progression-free interval.(**h-j**) Using GMS to perform prognostic stratification on methylation cluster 1 in Ricketts' study.
